# No increased risk of mature B-cell non-Hodgkin lymphoma after Q fever detected: results from a 16-year ecological analysis of the Dutch population incorporating the 2007–2010 Q fever outbreak

**DOI:** 10.1093/ije/dyac053

**Published:** 2022-03-30

**Authors:** Jesper M Weehuizen, Sonja E van Roeden, Sander J Hogewoning, Wim van der Hoek, Marc J M Bonten, Andy I M Hoepelman, Chantal P Bleeker-Rovers, Peter C Wever, Jan Jelrik Oosterheert

**Affiliations:** Department of Internal Medicine and Infectious Diseases, University Medical Centre Utrecht and Utrecht University, Utrecht, Netherlands; Department of Internal Medicine and Infectious Diseases, University Medical Centre Utrecht and Utrecht University, Utrecht, Netherlands; Netherlands Comprehensive Cancer Organisation, Utrecht, Netherlands; Centre for Infectious Disease Control, National Institute for Public Health and the Environment, Bilthoven, Netherlands; Department of Medical Microbiology, University Medical Centre Utrecht and Utrecht University, Utrecht, Netherlands; Department of Internal Medicine and Infectious Diseases, University Medical Centre Utrecht and Utrecht University, Utrecht, Netherlands; Department of Internal Medicine and Infectious Diseases, Radboud University Medical Centre and Radboud Expertise Centre for Q fever, Nijmegen, Netherlands; Department of Medical Microbiology and Infection Control, Jeroen Bosch Hospital, ’s-Hertogenbosch, Netherlands; Department of Internal Medicine and Infectious Diseases, University Medical Centre Utrecht and Utrecht University, Utrecht, Netherlands

**Keywords:** mature B-cell non-Hodgkin lymphoma, Q fever, *Coxiella burnetii*, Dutch Q fever outbreak, lymphomagenesis

## Abstract

**Background:**

A causative role of *Coxiella burnetii* (the causative agent of Q fever) in the pathogenesis of B-cell non-Hodgkin lymphoma (NHL) has been suggested, although supporting studies show conflicting evidence. We assessed whether this association is present by performing a detailed analysis on the risk of mature B-cell NHL after Q fever during and after the largest Q fever outbreak reported worldwide in the entire Dutch population over a 16-year period.

**Methods:**

We performed an ecological analysis. The incidence of mature B-cell NHL in the entire Dutch population from 2002 until 2017 was studied and modelled with reported acute Q fever cases as the determinant. The adjusted relative risk of NHL after acute Q fever as the primary outcome measure was calculated using a Poisson regression.

**Results:**

Between January 2002 and December 2017, 266 050 745 person-years were observed, with 61 424 diagnosed with mature B-cell NHL. In total, 4310 persons were diagnosed with acute Q fever, with the highest incidence in 2009. The adjusted relative risk of NHL after acute Q fever was 1.02 (95% CI 0.97–1.06, *P* = 0.49) and 0.98 (95% CI 0.89–1.07, *P* = 0.60), 0.99 (95% CI 0.87–1.12, *P* = 0.85) and 0.98 (95% 0.88–1.08, *P* = 0.67) for subgroups of diffuse large B-cell lymphoma, follicular lymphoma or B-cell chronic lymphocytic leukaemia, respectively. Modelling with lag times (1–4 years) did not change interpretation.

**Conclusion:**

We found no evidence for an association between *C. burnetii* and NHL after studying the risk of mature B-cell NHL after a large Q fever outbreak in Netherlands.

Key Messages

- We found no increased risk of mature B-cell NHL after a large Q fever outbreak in the entire Dutch population.
- There was no change in the conclusion after modelling with different subgroups of mature B-cell NHL and different lag times.
- The lack of an increased risk of mature B-cell NHL after Q fever makes an association with *Coxiella burnetii* unlikely.

## Introduction

Non-Hodgkin lymphoma (NHL) is among the most frequently diagnosed cancers in the Western world.^1^ The aetiology of NHL is believed to be multifactorial and besides toxic environmental factors such as alcohol, smoking, chemicals and genetic predisposition,^1^ several microbial pathogens (e.g. *Borrelia burgdorferi*, human immunodeficiency virus, Epstein-Barr virus)^2–4^ have been associated with an increased risk of NHL.

Netherlands faced the largest outbreak of Q fever ever recorded between 2007 and 2010. The outbreak occurred in a densely populated area where intense farming is practised.^5^ During the outbreak >4000 acute Q fever cases were reported. However, seroprevalence studies estimated a 50- to 75-fold excess of patients actually infected with *Coxiella burnetii*.^6^ The implementation of restrictive farming measures ended the outbreak.^5^

In 2016, it was suggested that *C. burnetii*, the bacterial agent causing Q fever, could also be added to the list of bacteria causing B-cell NHL. A French study found seven B-cell NHL cases in 1468 Q fever patients referred to a tertiary Q fever centre, which translated into a 25-fold and 7-fold risk of diffuse large B-cell lymphoma and follicular lymphoma compared with the general French population.^7^ Overproduction of interleukin-10 (IL-10), an inflammatory cytokine associated with NHL,^8^ was observed in Q fever patients developing lymphoma, which was hypothesized to be the underlying mechanism of lymphomagenesis.^7^ In Netherlands, one study using two different analyses assessed the possible association between NHL and *C. burnetii*. First, the incidence of NHL before and after the Dutch Q fever outbreak was studied, which did not confirm the association.^9^ The second analysis was performed in 439 chronic Q fever patients, of whom five patients had NHL, and reported a 5-fold risk of NHL compared with the general population. B-cell chronic lymphocytic leukaemia (60%) was the most predominant NHL in the Dutch chronic Q fever patients.^9^

A recent review on Q fever considered it proven that infection with *C. burnetii* is associated with an increased risk of B-cell NHL,^10^ although this is still widely debated. Risks of selection and detection bias were present in the analysis of both the Dutch chronic Q fever patients and the tertiary referred French Q fever patients.^7^^,^^9^ In addition, several conducted studies did not support the claim. The analysis in the entire Dutch population did not show an increased risk of NHL after Q fever.^9^ The detection of *C. burnetii* in lymphoma tissue was deemed a non-specific finding since the bacterium could also be demonstrated in tissue from non-NHL controls,^11^ and HIV patients with and without NHL had similar rates of infection with *C. burnetii*.^12^ Current literature therefore does not give us clear answers on the association between *C. burnetii* and NHL.

The previously mentioned Dutch population-based analysis of NHL incidence used crude proxy parameters to classify areas with high, intermediate and low Q fever endemicity as determinant, potentially leading to misclassification bias.^9^ The current study aims to provide a clear answer on the association *between C. burnetii* and B-cell NHL by conducting another study in the Dutch population. We matched data on the incidence of mature B-cell NHL more precisely with improved data on the incidence of Q fever in the Dutch population during and after the Q fever outbreak, instead of using a proxy parameter. Therefore, the risk of misclassification bias is minimized.

## Methods

### Study design

We performed an ecological analysis. The incidence of mature B-cell NHL in the entire Dutch population from 2002 until 2017 was studied, modelled with reported acute Q fever cases as the determinant. The outcome measure was the adjusted relative risk of NHL after acute Q fever. The hypothesis was that the incidence of NHL is increased in people with a history of a documented acute Q fever episode.

### Inclusion of patients and data collection

Acute Q fever notifications were provided by the National Institute for Public Health and Environment (RIVM). RIVM registers contained all medically diagnosed acute Q fever cases as reporting of laboratory-confirmed acute Q fever is obligatory by law in Netherlands. Data were provided on an individual level with the four-digit postal code area of residency, date of notification, month and year of birth, and gender.

Numbers of newly diagnosed mature B-cell NHL were collected from the Netherlands Cancer Registry (NCR), managed by the Netherlands Comprehensive Cancer Organization (IKNL). The IKNL systematically reviews medical charts in each hospital and registers all *new* cases of malignancy in Netherlands. Data were shared on an individual level, including the four-digit postal code area of residency, date of diagnosis, full date of birth and gender.

Statistics Netherlands (CBS) provided the number of inhabitants of each four-digit postal code area. There are ∼4000 four-digit postal code areas in Netherlands, with slight variation and change over time. Probabilistic record linkage was performed in the secure environment of the CBS. The CBS grouped the Dutch population by calendar year, 10-year age category, gender and four-digit postal code area. The number of person-years (PY) for each subgroup was provided. Furthermore, these data were combined with the number of acute Q fever notifications and NHL diagnosis in every group. Therefore, incidence rates for acute Q fever and NHL could be calculated for all small subgroups (per four-digit postal code area, 10-year age category, gender and year of occurrence). For some cases (1.73%) linkage was not successful due to missing data. These cases were not included in the analysis.

### Definitions

National notification criteria for acute Q fever were modified several times during the large 2007–2010 Q fever outbreak in Netherlands, but always consisted of clinical criteria (pneumonia, or hepatitis, or fever) and a laboratory confirmation.^13^ Mature B-cell lymphoma were defined according to the 2016 revision of the World Health Organization classification of lymphoid neoplasms.^14^ Plasma cell myeloma were not included in the data. The different subtypes of B-cell neoplasms with ICD-O-3 codes (International Classification of Diseases for Oncology, third edition) are displayed in the Supplementary material Section 1 (available as Supplementary data at *IJE* online).^15^

### Statistics

Data from the CBS, NCR and RIVM were stored in Microsoft Excel (Windows version 2010). Data analysis was performed in R studio (version 3.6.2). SPSS (version 25.0) and Microsoft Excel (Windows version 2013) were used for generating figures. Since multiple B-cell NHL can occur within one individual, patients were not censored after developing their first NHL. A second primary NHL was scored as a new lymphoma in the analysis. Crude absolute risks (or incidence rates) were calculated for the Dutch population grouped by calendar year, 10-year age category, gender and four-digit postal code area. Our outcome measure was the adjusted relative risk of mature B-cell NHL, with the number of acute Q fever cases modelled as the determinant of interest. The natural logarithm of the number of PY at risk of the outcome was included in the models as an offset. Age, gender and year were entered as covariables in the model. Moreover, an interaction between age and gender was entered in the model. The model with interaction had a lower Akaike Information Criterion compared with the model without interaction (likelihood ratio test). To account for the possibility of a delayed effect for an increased risk of B-cell NHL after Q fever, models were fitted with lag times (0–4 years). Lag times of >4 years were considered to be unlikely based on data describing the time between exposure to Q fever infection and the occurrence of NHL.^7^^,^^9^^,^^11^^,^^16^^,^^17^ Model assumptions (mean and variance equality in the model) were verified by calculating phi. The calculated phi’s were between 1.06 and 1.07 for the models with different lag times, indicating Poisson distribution. Model diagnostics of the final model were performed by checking residual plots and influential observations. We reported relative risks (incidence rate ratio’s) with 95% CIs (Wald) and *P*-values. Finally, Poisson regression models were fitted for specific subgroups of NHL: diffuse large B-cell lymphoma, B-cell chronic lymphocytic leukaemia and follicular lymphoma. These subgroups were selected beforehand based on previous publications, suggesting a specifically increased risk for those types of NHL.^7^^,^^9^ Models were again fitted with lag times (1–4 years) and similar model covariables.

## Results

**Figure 1:**
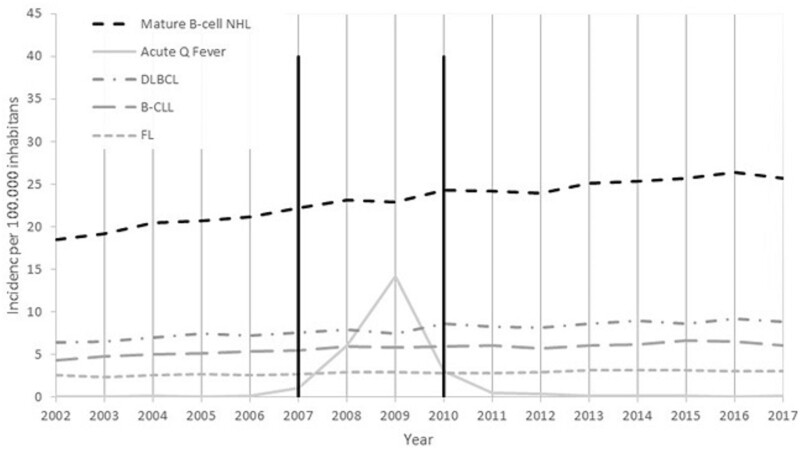
Absolute incidence of mature B-cell NHL and acute Q fever per year. The Q fever outbreak occurred from 2007 to 2010 and is marked between the black lines. NHL, non-Hodgkin lymphoma; DLBCL, diffuse large B-cell lymphoma; FL, follicular lymphoma; B-CLL, B-cell chronic lymphocytic leukaemia.

**Figure 2:**
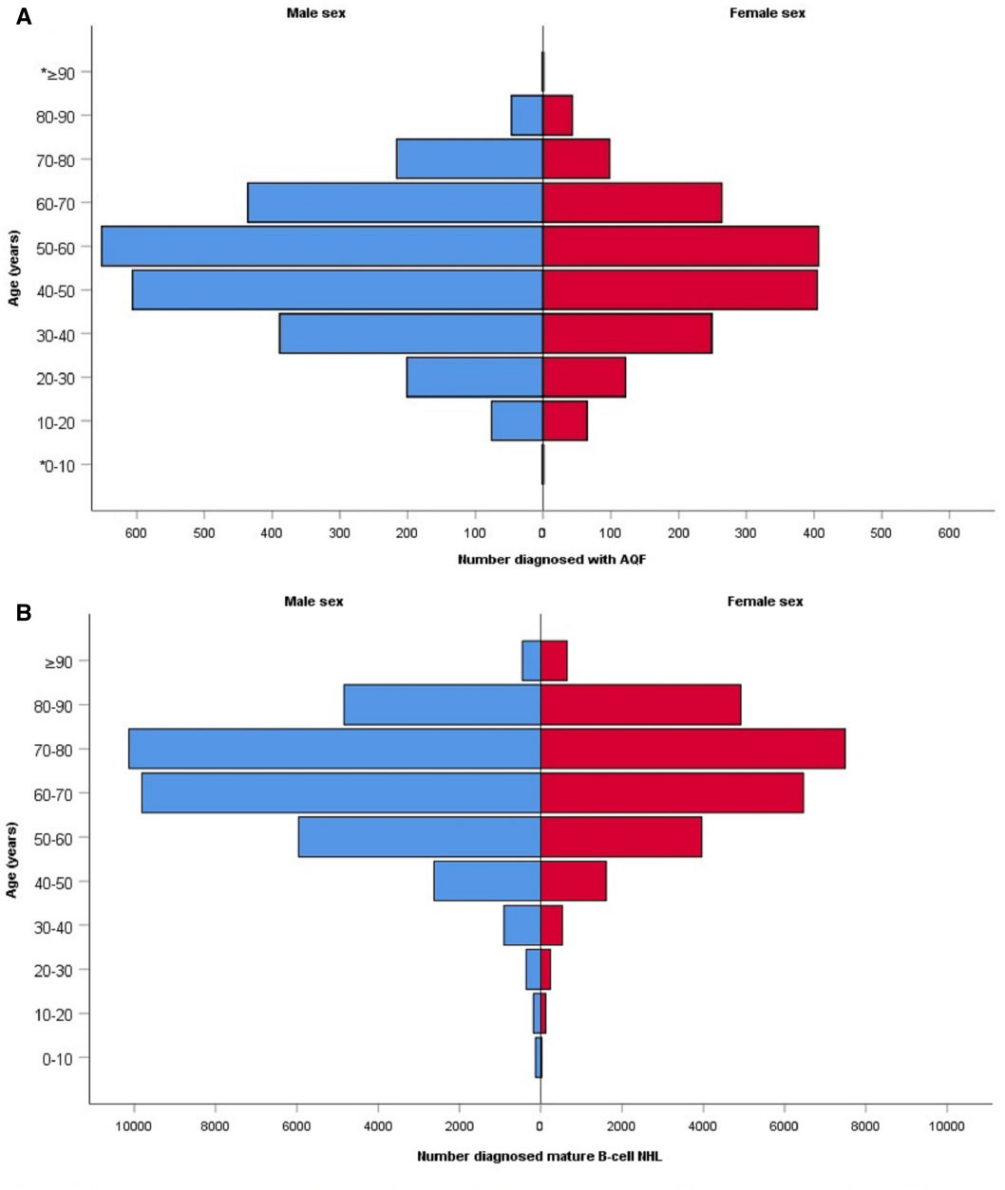
Number of people with acute Q fever and mature B-cell NHL per 10-year age category and sex. AQF, acute Q fever; NHL, non-Hodgkin lymphoma. *The number of people diagnosed with AQF is <10 in these categories and are not displayed in this figure due to privacy regulations form Statistics Netherlands (CBS).

Between the start of 2002 and the end of 2017, 266 050 745 PY were observed in Netherlands with a mean population size of 16.5 million inhabitants per year. During these 16 years of follow-up 61 424 persons were diagnosed with mature B-cell NHL. In 320 persons (0.5%) a second primary NHL was diagnosed. The crude incidence rate of NHL in this period was 22.8 per 100 000 PY. The yearly incidence ranged between 18.5 and 26.4 cases per 100 000 PY, with the highest incidence in 2016. The most frequent NHL subtypes diagnosed were diffuse large B-cell lymphoma, B-cell chronic lymphocytic leukaemia and follicular lymphoma, with an incidence rate of 7.9, 5.7 and 2.8 per 100 000 PY. In total, 4310 persons were diagnosed with acute Q fever between 2002 and 2017 (Table 1). For Q fever, the yearly incidence rate reached its peak in 2009 with 14.2 cases per 100 000 PY. In 2002 and 2003 no persons were diagnosed with acute Q fever (Figure 1). Both NHL and Q fever are more frequently diagnosed in men. Furthermore, the highest incidence for NHL occurred between ages 60 and 80 years, and between ages 40 and 60 years for Q fever (Figure 2).

**Table 1:** Number of diagnosed mature B-cell non-Hodgkin lymphoma and acute Q fever in the Dutch population between 2002 and 2017

	Population (PY)	AQF		Mature B-cell NHL							
				Total		DLBCL		B-CLL		FL	
		Number	Inc.	Number	Inc.	Number	Inc.	Number	Inc.	Number	Inc.
**Total**	266 050 745	4310	1.6	61 424	22.8	21 097	7.9	15 111	5.7	7562	2.8
**Male (%)**	131 726 100 (49.5%)	2646 (61.4%)	2.0	35 403 (57.6%)	26.8	11 608 (55.0%)	8.8	9285 (61.4%)	7.0	3861 (51.1%)	2.9
**Female (%)**	134 324 645 (50.5%)	1664 (38.6%)	1.2	26 021 (42.4%)	19.5	9489 (45.0%)	7.1	5826 (38.6%)	4.3	3701 (48.9%)	2.8

Inc., incidence reported as cases per 100 000 person-years. NHL, non-Hodgkin lymphoma; PY, person-years; AQF, acute Q fever; DLBCL, diffuse large B-cell lymphoma; FL, follicular lymphoma; B-CLL, B-cell chronic lymphocytic leukaemia.

The relative risks (RRs) for mature B-cell NHL after acute Q fever are shown in Table 2. RRs are calculated for all mature B-cell NHL and the three largest subgroups. In the model without lag time, the RR for all NHL was 1.02 (95% CI 0.97–1.06, *P* = 0.49). For the subgroups diffuse large B-cell lymphoma, follicular lymphoma and B-cell chronic lymphocytic leukaemia, RRs were 0.98 (95% CI 0.89–1.07, *P* = 0.60), 0.99 (95% CI 0.87–1.12, *P* = 0.85) and 0.98 (95% CI 0.88–1.08, *P* = 0.67), respectively. RR for NHL after acute Q fever modelled with different lag times (1–4 years), which showed no association either, are also shown in Table 2.

**Table 2:** Relative risk of mature B-cell non-Hodgkin lymphoma (all subtypes)

	All mature B-cell NHL		DLBCL		FL		B-CLL	
Estimates	RR (95% CI)	*P*-value	RR (95% CI)	*P*-value	RR (95% CI)	*P*-value	RR (95% CI)	*P*-value
**0-year lag time**	1.02 (0.97–1.06)	0.49	0.98 (0.89–1.07)	0.60	0.99 (0.87–1.12)	0.85	0.98 (0.88–1.08)	0.67
**1-year lag time**	1.00 (0.95–1.05)	0.94	1.00 (0.92–1.08)	0.98	0.93 (0.80–1.08)	0.37	1.00 (0.91–1.10)	0.96
**2-year lag time**	0.99 (0.95–1.04)	0.70	1.00 (0.92–1.08)	0.91	0.93 (0.80–1.08)	0.34	0.99 (0.90–1.09)	0.92
**3-year lag time**	1.00 (0.95–1.04)	0.88	1.03 (0.59–1.10)	0.44	0.98 (0.87–1.11)	0.80	0.92 (0.82–1.04)	0.18
**4-year lag time**	1.01 (0.97–1.06)	0.56	1.03 (0.97–1.11)	0.34	0.95 (0.83–1.10)	0.50	1.01 (0.93–1.10)	0.79

DLBCL, FL and B-CLL by different lag times. Analyses were adjusted for age, gender and time. NHL, non-Hodgkin lymphoma; DLBCL, diffuse large B-cell lymphoma; FL, follicular lymphoma; B-CLL, B-cell chronic lymphocytic leukaemia; RR, relative risk.

## Discussion

We found no increased risk of mature B-cell NHL after Q fever in the entire Dutch population with 16 years of follow-up, during and after the largest Q fever outbreak ever reported. Previous studies reported contradictory outcomes on this association. A French study group reported a 9-fold increased risk of B-cell NHL after Q fever, based on 7 B-cell NHL cases in a cohort of 1468 Q fever patients, compared with the general French population.^7^ Another study reported a 5-fold increase in the risk of NHL in chronic Q fever patients, based on 5 NHL cases in 439 chronic Q fever patients.^9^ In the same article, an analysis in the entire Dutch population found no evidence for an increased risk of NHL after Q fever, in spite of a temporary increase in NHL in 2009 in areas with high endemicity of Q fever compared with low endemic areas.^9^ Yet, all three analysis suffered from serious methodological issues resulting in a risk of biased estimates. The French study was subject to selection bias and detection bias, since all Q fever patients were selected from a tertiary referral centre.^7^ The risk of B-cell NHL in a population referred to a (Q fever) tertiary referral centre is presumably higher than the risk of B-cell NHL in the general population. The patients in the Dutch chronic Q fever database were extensively examined with positron emission tomography–computed tomography (PET-CT) and laboratory investigations, which potentially led to detection bias.^9^ In the analysis of the entire Dutch population, the probability of selection bias was reduced since it was population-based.^9^ However, there was a risk of misclassification of exposure. The association was investigated by using a proxy parameter, dividing Netherlands into three areas, based on the number of reported acute Q fever cases (0, 1 or >1) reported between 2007 and 2010. More detailed data were not available to the researchers for privacy reasons. Besides the fact that it was uncertain whether individuals were actually exposed, categories were based on the pooled number of Q fever cases over a 4-year period, which could have influenced the association and distorted the results in both directions.

In this study, data from the entire population were used, reducing the chance of the earlier-mentioned selection bias. Furthermore, we included data over a 16-year period. After being diagnosed with acute Q fever, it is possible that patients are monitored more closely after enduring acute Q fever, which can lead to a possible earlier diagnosis of mature B-cell NHL. However, the risk of detection bias will be minimal. The ‘missed’ NHL in the rest of the population will probably still be diagnosed during the study period and is further reduced by modelling the risk of NHL after Q fever with different lag times. In addition to the previous population-based study, we have reduced the risk of misclassification of exposure to Q fever by probabilistic record linkage on a highly detailed level. Moreover, in Netherlands, Q fever is a notifiable disease. Therefore, all known Q fever cases have been identified in this study. All new cancer diagnoses are registered by the NCR, by systematically reviewing all medical charts in Netherlands. Both registries, therefore, provide a solid base to assess the presence of an association between Q fever and NHL. Furthermore, the duration of follow-up after the end of the Dutch Q fever outbreak was 7 years. Since the effect of Q fever on the risk of NHL could be delayed, lag times were modelled, which did not alter any of the conclusions. Finally, specific subgroups of interest based on previous studies were analysed separately. No association in those subgroups was present either.

Our study suffers from several limitations. Based on serological screening studies, it is estimated that 40 000–50 000 people were infected with *C. burnetii* during the outbreak between 2007 and 2010.^6^ This implies that the number of reported cases (*N* = 4310) is an underestimation of the actual number of infected individuals, as many patients have not been formally diagnosed during their episode of acute Q fever because the disease is often asymptomatic. However, the number of reported cases is most likely a good predictor of the number of actual cases in certain areas.^18^ It is possible that the number of reported Q fever cases in high endemic areas relatively increased over time during the outbreak due to increased awareness amongst clinicians in those areas in comparison to low endemic areas. On the other hand, it is also possible that in high endemic areas the role of asymptomatic and missed Q fever cases is higher due to the high circulation of *C. burnetii.* Therefore, we conducted two sensitivity analysis by performing the analysis after doubling the Q fever cases in the three most affected provinces in the south-east of Netherlands and on the other hand after doubling the number of Q fever cases in the nine least affected provinces. This sensitivity analysis did not lead to another conclusion. The results are displayed in the Supplementary material Section 2 (available as Supplementary data at *IJE* online). Another limitation is the fact that we have not accounted for migration. Data were analysed grouped by the CBS per four-digit postal code area. Individuals moving to different postal code areas, with and without the outcome of interest, may still cause misclassification of exposure risk in the data set. However, this misclassification has an effect on the estimate when a person moves to another postal code area during the lag time between a *C. burnetii* infection and diagnosis of mature B-cell NHL. Therefore, the effect on the estimates will probably be small. Finally, in the sensitivity analyses in which lag times were introduced, grouping of individuals within age categories will be less accurate. Ageing of individuals is not accounted for and may cause misclassification as well. However, it is not expected that this misclassification occurs more in patients with or without the exposure and therefore has a small effect on the estimate.

In a recent review on Q fever, it is hypothesized that the association between Q fever and B-cell NHL would be most relevant for chronic Q fever.^10^ In the current study, chronic Q fever patients have not been studied separately. The study by van Roeden *et al.* evaluated the incidence of NHL in the Dutch national chronic Q fever database earlier.^9^ In the current study, we analysed the association between Q fever and NHL with lag times of ≤4 years. Since the mean time between acute Q fever and NHL is 8 months and the median time between acute Q fever and diagnosis of chronic Q fever is 13 months,^19^ the hypothetical excess risk of NHL in chronic Q fever patients is included in the current analysis.

In conclusion, no increased risk of mature B-cell NHL after Q fever was found in the entire Dutch population that suffered the largest Q fever outbreak ever recorded. This makes the presence of a clinically relevant association between *C. burnetii* and NHL highly unlikely.

## Ethics approval

The CBS has a legal mandate to collect and analyse individual-level data.^20^ Data were only accessible in a remote access environment of the CBS to the corresponding and second author, who signed a confidentiality statement. Data analyses were performed in this remote environment and all exported output was untraceable to individuals (numbers <10 were not allowed as export in tables). Output was checked by a privacy controller of the CBS. Since all data were anonymized and participants were not subject to any interventions as part of this study, we did not need to obtain approval from a medical ethics committee according to Dutch legislation.

## Data availability

The data collected for this study will not be made available to others. Data were only available in a remote access environment of the CBS due to privacy regulations.

## Supplementary data

Supplementary data are available at *IJE* online.

## Author contributions

J.M.W. contributed to conceptualization, data curation, formal analysis, methodology, project administration, visualization, writing—original draft, and review and editing. S.E.v.R. contributed to conceptualization, funding acquisition, data curation, formal analysis, methodology, project administration, visualization, writing—original draft, and review and editing. S.J.H. and W.v.d.H. contributed to conceptualization, funding acquisition, data curation, formal analysis, methodology, writing—review and editing. M.J.M.B. contributed to formal analysis, methodology, writing—review and editing. C.P.B.-R. and P.C.W. contributed to conceptualization, funding acquisition, formal analysis, methodology, writing—review and editing, and supervision. A.I.M.H. and J.J.O. contributed to conceptualization, funding acquisition, formal analysis, methodology, project administration, writing—review and editing, and supervision.

## Funding

This work was supported financially by ZonMW (grant no. 50–52200-98–807). The funder had no role in study design, data collection, data analysis, data interpretation or writing of the report.

## Conflict of interest

None declared.

## Supplementary Material

dyac053_Supplementary_Data
