# Supplementary material for: No increased risk of mature B-cell non-Hodgkin lymphoma after Q fever detected: results from a 16-year ecological analysis of the Dutch population incorporating the 2007–2010 Q fever outbreak
Source: Int J Epidemiol. 2022 Mar 30;51(5):1481–8. doi: 10.1093/ije/dyac053 (PMC9557853; doi:10.1093/ije/dyac053)
Supplement: dyac053_Supplementary_Data [file dyac053_supplementary_data.docx]

**Supplementary material section 1**

| Table 3  Number of mature B-cell NHL subtypes with ICD-O-3 codes in the Dutch population | | | |
| --- | --- | --- | --- |
| Mature B-cell NHL subtypes | **ICD-O-3 codes** | **Number** | **Incidence** |
| B-CLL | 9820, 9823 | 15,111 | 5.68 |
| B-PLL | 9832, 9833 | 142 | 0.05 |
| Burkitt Lymphoma/leukemia | 9687, 9826 | 826 | 0.31 |
| DLBCL and other variants | 9675, 9678, 9679, 9680, 9684, 9688, 9712, 9735, 9737, 9738, 9971 | 21,097 | 7.92 |
| Follicular lymphoma | 9690, 9691, 9695, 9698 | 7,562 | 2.84 |
| ‘Hairy cell’ leukemia | 9940 | 1,070 | 0.40 |
| Lymfoplasmacytic lymphoma / M. Waldeström | 9671, 9761 | 3,853 | 1.45 |
| Mantel cell lymphoma | 9873 | 2,880 | 1.08 |
| Marginal zone lymphoma (MALT/BALT/SALT) and immunoprolifaratic disorder of the small intestines | 9689, 9699, 9764 | 3,806 | 1.43 |
| Other and unspecified B-cell NHL | 9590, 9591, 9596, 9762 | 1,930 | 0.73 |
| Small cell B-cell lymphoma | 9670 | 3,147 | 1.19 |
| Total number of mature B-cell NHL |  | **61,424** | **22.8** |
| Incidence = cases per 100,000 person years.  NHL=non-Hodgkin lymphoma. ICD-O-3= International Classification of Diseases for Oncology, third edition. B-CLL=B-cell chronic lymphocytic leukemia. B-PLL=B-cell prolymphocytic leukaemia. DLCBL=diffuse large cell B-cell lymphoma. MALT=mucosa-associated lymphoid tissue. BALT= bronchus-associated lymphoid tissue lymphoma. SALT= Skin-Associated Lymphoid Tissue | | | |

**Supplementary material section 2**

| Table 4a  (Sensitivity analysis: Number of Q fever notifications multiplied by 2 in each postal code area in the three provinces in the southeast of the Netherlands most affected by the Q fever outbreak)  Relative risk for mature B-cell NHL (all subtypes) DLCBL, FL and B-CLL by different lagtimes | | | | | | | | |
| --- | --- | --- | --- | --- | --- | --- | --- | --- |
|  | All mature B-cell NHL |  | DLCBL |  | FL |  | B-CLL |  |
| Estimates | RR (95% CI) | P value | RR (95% CI) | P value | RR (95% CI) | P value | RR (95% CI) | P value |
| 0 year lagtime | 1.01 (0.98 – 1.03) | 0.58 | 0.99 (0.94 – 1.03) | 0.53 | 0.99 (0.93 – 1.06) | 0.77 | 0.98 (0.93 – 1.04) | 0.59 |
| 1 year lagtime | 1.00 (0.98 – 1.02) | 1.00 | 1.00 (0.96 – 1.04) | 0.92 | 0.95 (0.88 – 1.04) | 0.26 | 1.00 (0.96 – 1.05) | 0.93 |
| 2 year lagtime | 1.00 (0.97 – 1.02) | 0.70 | 1.00 (0.96 – 1.04) | 0.84 | 0.96 (0.89 – 1.04) | 0.32 | 1.00 (0.95 – 1.05) | 0.97 |
| 3 year lagtime | 1.00 (0.97 – 1.02) | 0.68 | 1.01 (0.97 – 1.05) | 0.68 | 0.99 (0.93 – 1.06) | 0.78 | 0.96 (0.90 – 1.02) | 0.20 |
| 4 year lagtime | 1.00 (0.98 – 1.03) | 0.71 | 1.02 (0.98 – 1.05) | 0.39 | 0.98 (0.91 – 1.05) | 0.51 | 1.00 (0.96 – 1.05) | 0.83 |
| Analysis were adjusted for age, gender and time.  NHL=non-Hodgkin lymphoma. DLCBL=diffuse large cell B-cell lymphoma. FL=follicular lymphoma. B-CLL=B-cell chronic lymphocytic leukemia. RR=relative risk. CI=confidence interval | | | | | | | | |

| Table 4b  (Sensitivity analysis: Number of Q fever notifications multiplied by 2 in postal code each area in the nine provinces of the Netherlands least affected by the Q fever outbreak)  Relative risk for mature B-cell NHL (all subtypes) DLCBL, FL and B-CLL by different lagtimes | | | | | | | | |  |
| --- | --- | --- | --- | --- | --- | --- | --- | --- | --- |
|  | All mature B-cell NHL |  | DLCBL |  | FL |  | B-CLL |  | |
| Estimates | RR (95% CI) | P value | RR (95% CI) | P value | RR (95% CI) | P value | RR (95% CI) | P value | |
| 0 year lagtime | 1.02 (0.98 – 1.06) | 0.36 | 0.99 (0.91 – 1.07) | 0.72 | 1.00 (0.90 – 1.12) | 0.99 | 0.99 (0.90 – 1.08) | 0.84 | |
| 1 year lagtime | 1.00 (0.96 – 1.05) | 0.84 | 1.00 (0.92 – 1.07) | 0.92 | 0.97 (0.86 – 1.10) | 0.63 | 0.99 (0.90 – 1.08) | 0.77 | |
| 2 year lagtime | 0.99 (0.95 – 1.04) | 0.73 | 1.00 (0.93 – 1.07) | 0.98 | 0.94 (0.82 – 1.08) | 0.38 | 0.99 (0.91 – 1.08) | 0.83 | |
| 3 year lagtime | 1.01 (0.97 – 1.05) | 0.74 | 1.05 (0.98 – 1.11) | 0.15 | 0.99 (0.88 – 1.11) | 0.83 | 0.92 (0.83 – 1.03) | 0.16 | |
| 4 year lagtime | 1.02 (0.98 – 1.06) | 0.36 | 1.04 (0.97 – 1.10) | 0.27 | 0.96 (0.85 – 1.09) | 0.51 | 1.01 (0.93 – 1.10) | 0.71 | |
| Analysis were adjusted for age, gender and time.  NHL=non-Hodgkin lymphoma. DLCBL=diffuse large cell B-cell lymphoma. FL=follicular lymphoma. B-CLL=B-cell chronic lymphocytic leukemia. RR=relative risk. CI=confidence interval | | | | | | | | |  |
